# Discovery of Benzopyrrolizidines as Promising Antigiardiasic Agents

**DOI:** 10.3389/fcimb.2021.828100

**Published:** 2022-01-12

**Authors:** Juan Carlos Auriostigue-Bautista, Eduardo Hernández-Vázquez, David González-Calderón, Jorge Luís Figueroa-Romero, Adriana Castillo-Villanueva, Angélica Torres-Arroyo, Martha Ponce-Macotela, Yadira Rufino-González, Mario Martínez-Gordillo, Luis D. Miranda, Jesús Oria-Hernández, Horacio Reyes-Vivas

**Affiliations:** ^1^ Laboratorio de Bioquímica-Genética, Instituto Nacional de Pediatría. Insurgentes Sur 3700-C, Col. Insurgentes Cuicuilco, Alcaldía Coyoacán, Ciudad de México, Mexico; ^2^ Departamento de Química Orgánica, Instituto de Química, Universidad Nacional Autónoma de México, Circuito Exterior, Ciudad Universitaria, Alcaldía Coyoacán, Ciudad de México, Mexico; ^3^ Laboratorio de Parasitología-Experimental, Instituto Nacional de Pediatría. Insurgentes Sur 3700-C, Col. Insurgentes Cuicuilco, Alcaldía Coyoacán, Ciudad de México, Mexico

**Keywords:** Giardia, antigiardiasic, benzopyrrolizidine, chemical library, drug design, chemotherapy

## Abstract

Current treatments for giardiasis include drugs with undesirable side effects, which increase the levels of therapeutic desertion and promote drug resistance in the parasites. Herein, we describe the antigiardiasic evaluation on Giardia lamblia trophozoites of a structurally diverse collection of 74 molecules. Among these scaffolds, we discovered a benzopyrrolizidine derivative with higher antigiardiasic activity (IC_50_ = 11 µM) and lower cytotoxicity in human cell cultures (IC_50_ = 130 µM) than those displayed by the current gold-standard drugs (metronidazole and tinidazole). Furthermore, this compound produced morphologic modifications of trophozoites, with occasional loss of one of the nuclei, among other changes not observed with standard giardicidal drugs, suggesting that it might act through a novel mechanism of action.

## Introduction

Giardiasis, the infection caused by *Giardia lamblia*, persists as the most common enteropathogenic infection in humans, labeling it as a public health problem. Giardiasis has a worldwide distribution where resource-limited countries exhibit the highest prevalence rates (20-40%) (Baldursson and Karanis, 2011) . Children younger than 10 years (Cacciò and Ryan, 2008), older people, and immunocompromised groups are mostly affected. Giardiasis displays a wide variety of clinical manifestations; the chronic disease may cause malnutrition and failure to thrive syndrome, having a marked impact in the physical and intellectual cognitive development in pediatric patients (Farthing, 1997; Baldursson and Karanis, 2011). Furthermore, the assemblages A and B of *G. lamblia* (Syn. *G. intestinalis* or *G. duodenalis*) are zoonotic (Thompson and Ash, 2019), therefore, it has been suggested that both domestic and farm animals can play a role as reservoirs for this parasite (Sahraoui et al., 2019).

Several compounds have been reported with antigiardiasic activity, classified as nitro-containing derivatives (nitroimidazoles, nitrothiazoles, and nitrofurans) (Miyamoto et al., 2013; Leitsch, 2019), benzimidazoles, quinacrine, paromomycin, and bacitracin (Leitsch, 2015; Argüello-García et al., 2020). Most of them have a limited effectivity and significant side effects, thus restraining their use (Bennett et al., 2015). Besides, emergent resistance in *Giardia* strains, which provoke the failure of treatments, has been reported (Nabarro et al., 2015; Carter et al., 2018). Therefore, efforts for searching new and more efficient giardicidal agents with better activity, low toxicity, and, consequently, fewer adverse side effects are of utmost importance.

In the last two decades, diversity-oriented synthesis (DOS) (Mukherjee et al., 2015), emerged as a reliable and successful strategy for achieving new scaffolds in the drug discovery field. This approach permits the exploration of chemical space through the construction of unique and varied scaffolds with high complexity and diversity (Yi et al., 2018). Indeed, the strategy has been satisfactorily applied in the search for new anti-HIV molecules (Shiroishi-Wakatsuki et al., 2019), compounds against schistosomiasis (Abdulla et al., 2009), as well as other examples (McLeod et al., 2015). In the present work we searched for novel antigiardiasic molecules by using a low-throughput screening approach on a chemical collection derived from different diversity-oriented programs. Our low-throughput screening campaign revealed a novel compound with high antigiardiasic activity and low cytotoxicity in human cells, operating through a possibly novel mechanism of action and perhaps, new molecular targets.

## Results and Discussion

### Chemical Collection

**Scheme 1:**
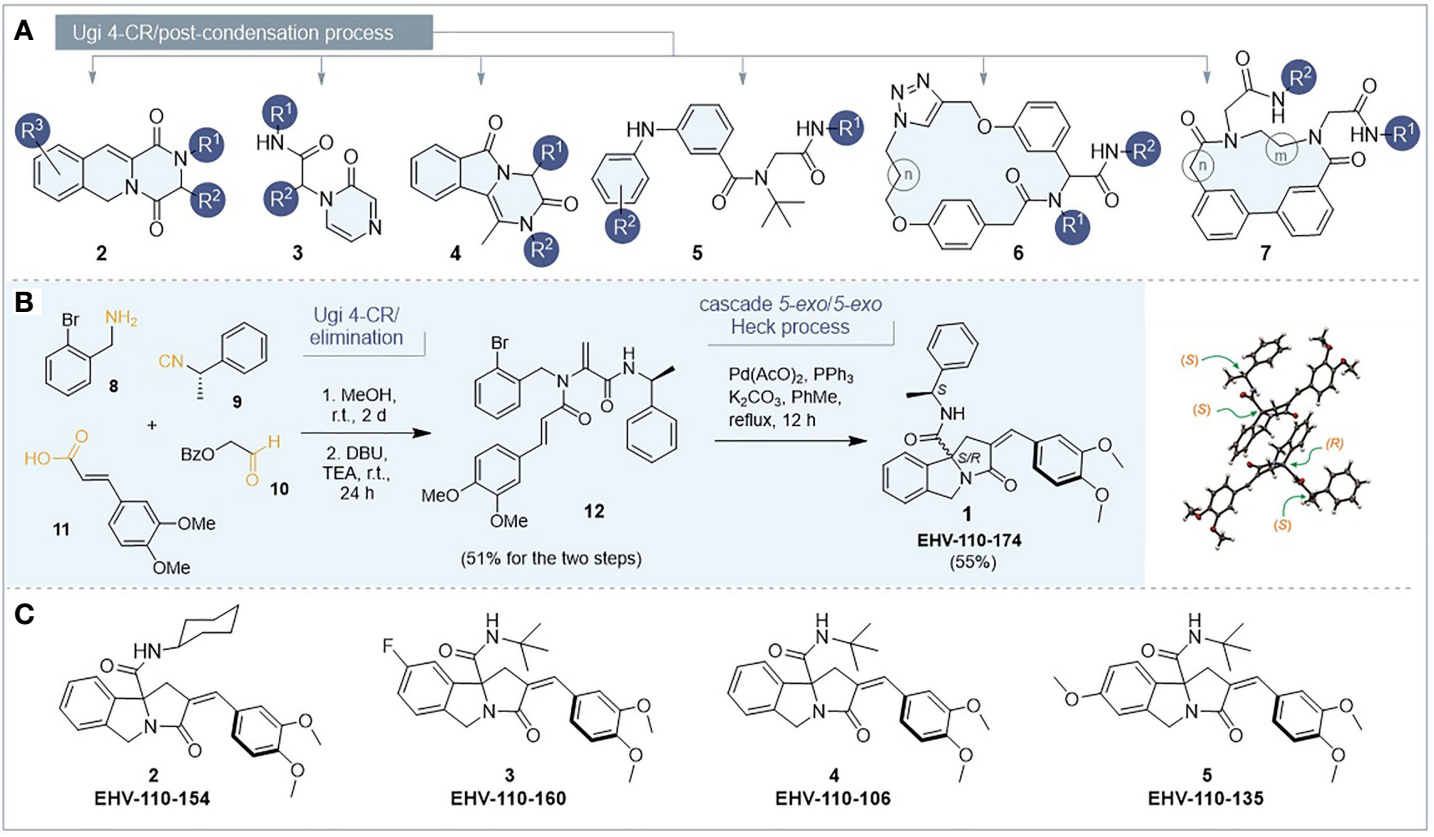
**(A)** General structures of the molecules in the collection. **(B)** Synthetic route for the preparation of benzopyrrolizidine **1** and X-ray structure; both diastereomers are shown (Thermal ellipsoids are drawn at 50% probability for all atoms except for hydrogen. CCDC Number = 2077691). **(C)** Pyrrolizidines for preliminary SAR.

The 74 compounds selected for the low-throughput screening campaign were obtained from our homemade collection and were prepared in accordance with previously reported works. The collection was divided into two sets. One set contained poly-azaheterocycles (**Scheme 1A**) such as benzopyrrolizidines (1) (Miranda and Hernández-Vázquez, 2015), pyrazinoisoquinolines (2) (Hernández-Vázquez and Miranda, 2016), peptidic pyrazinones (3) (Hernández-Vázquez et al., 2019), acyclic diphenylamines (4) (Chávez-Riveros et al., 2021), and isoindolones (5) (Icelo-Ávila et al., 2017). The second set consisted of *bis*(aryl ether)cyclophanes (6) (Hernández-Vázquez et al., 2018) and biphenyl-containing macrocycles (7) (Pérez-Labrada et al., 2017). The structures of the chemical collection are shown in Supporting Information (**Tables S1**–**S7**). The collection satisfied two criteria: high structural diversity and the presence of molecular scaffolds that do not resemble those compounds with previously reported antigiardiasic activity. It is worth mentioning that all the protocols commenced with a Ugi four-component reaction (Ugi-4CR), merged with further transformations. The Ugi-4CR combines an amine, a carboxylic acid, an aldehyde, and an isocyanide in a single step, allowing the preparation of dipeptides with several sites for further modifications. Thus, the proposed modular strategy allows the construction of collections with high structural diversity, in few synthetic steps (3-4 steps), from simple starting materials.

We show a representative example of the synthetic approach for the only new compound reported in this study, the benzopyrrolizidine 1 (EHV-110-174, **Scheme 1B**). This compound was prepared for two reasons: first, to verify if a pre-established stereogenic center could exert an asymmetric induction during the Heck tandem reaction; however, ^1^H-NMR spectrum (see *Supporting Information*) confirmed a 50:50 mixture. Additionally, the incorporation of a methyl group at the benzyl moiety present in EHV-110-174 may interact with key amino acids within the active site of the receptor, thus enhancing the antigiardiasic effect. This molecule was obtained by applying our reported 3-step protocol, from *o*-bromobenzylamine 8, (*S*)-(−)-α-methylbenzyl isocyanide 9, the aldehyde 10 and 3,4-dimethoxycinnamic acid 11. This input set of four-components was transformed into the dehydroalanine derivative 12 by an elimination process of the intermediate α-acylamino carboxamide. Then, a palladium-catalyzed *5-exo-trig/5-exo-trig* cascade assembled the benzopyrrolizidine system. The heterocycle was isolated as an inseparable diastereomeric mixture (*S,R* and *S,S*) in a global 28% yield. Crystallization, column chromatography and centrifugal thin layer chromatography with different solvent mixtures were tried for the separation, but all of them failed to yield pure samples. One of the asymmetric carbons came from the initial (*S*)-isocyanide, while the other one was constructed (in a ~50:50 ratio) by the first 5-*exo-trig* cyclization of the Heck tandem sequence. Efforts to separate both diastereomers were futile; however, we were able to obtain the appropriate crystals for X-ray analysis (CCDC Number = 2077691). Interestingly, we found that both diastereomers crystalized together in an unusual co-crystal (**Scheme 1B**). We are currently working on the separation of the diastereomeric mixture to investigate if the configuration of the quaternary carbon of the benzopyrrolizidine nucleus may influence the antigiardiasic activity and the results will be published later.

### Primary Molecular Screening

We evaluated the antigiardiasic activity from a first set of 54 molecules at 100 µM by using the cytotoxicity micro assay described under the Material and Methods section. Then, the viability was determined by reduction of XTT-tetrazolium salts to formazan. The first screening showed that five of the compounds induced death yields above 50% (**Table S8**). We then re-evaluated the same compounds by serial dilution method in the cytotoxicity microassay. At 50 µM, four compounds showed death yields above 80%; however, when they were tested at 12.5 µM, only one of them exhibited yields higher than 80% (EHV-110-174, **Table S9**). The lethality of EHV-110-174 remained above 80%, even at the lowest evaluated concentrations and was quite similar to that obtained with metronidazole (**Table S9**). We then evaluated a second set of 20 candidate molecules including both benzopyrrolizidines and *bis*(aryl ether)cyclophanes (**Table S10**). Those compounds showing death yields above 50% at 100 µM were also evaluated at 50 and 25 µM. The data showed one compound with the benzopyrrolizidine scaffold exhibiting death yields higher than 90% at 100 and 50 µM (EHV-110-135, 5). In contrast, only two macrocycles, EHV-110MC-60 and EHV-110MC-74 displayed 50% of death at 100 µM and 50 µM, respectively. Consequently, the molecules with a benzopyrrolizidine scaffold displayed the best antigiardiasic activity.

The IC_50_ values of the best antigiardiasic compounds were determined (**Table 1**). The compound EHV-110-174 had the lowest IC_50_ value (11 µM). This result is close to those for both tinidazole and metronidazole (**Table 1**). In contrast, the macrocyclic compound EHV-110MA-78 displayed the highest IC_50_ value (38 µM).

**Table 1:** IC_50_ values of best antigiardiasic compounds in trophozoites and HaCaT culture cells.

Compound	IC_50_ (µM)		Selectivity Index^3^
	Trophozoites	HaCaT	
**EHV-110-154^1^**	30	50.6 (4.1)	1.68
**EHV-110-160^1^**	20	141 (1.8)	7
**EHV-110-174^1^**	10.9 (1.7)	130 (4.8)	12
**EHV-110MA-78^2^**	38	86.9 (2.3)	2.3
Tinidazole	1.3 (0.3)	60.9 (5)	46
Metronidazole	2.6 (0.3)	71.86 (6.1)	27.6

Standard deviation values are in parenthesis.

^1^Benzopyrrolizidines.

^2^Bis(aryl ether) macrocycle.

^3^IC_50_ HaCaT/IC_50_Trophozoites.

To analyze the potential cytotoxicity in mammalian cells of compounds with the higher antigiardiasic activities, we determined the IC_50_ values in human keratinocytes cells (HaCaT), a cell line commonly used in cytotoxic assays (Lopes et al., 2016). The data showed that the macrocycle EHV-110MA-78 and the tricycle EHV-110-154 showed the lowest IC_50_ values (87 and 50 µM, respectively; **Table 1**). On the contrary, the lead compound EHV-110-174 and EHV-110-160, exhibited the highest IC_50_ values for HaCaT cells (130 and 141 µM, respectively), indicating low cytotoxicity against this cell line. Interestingly, both compounds also displayed the highest antigiardiasic activity. Furthermore, it is remarkable that EHV-110-174 showed less cytotoxicity than the current gold-standard drugs, tinidazole, and metronidazole, and with a Selectivity Index value of 12 (**Table 1**). It is worth noting that compounds based on the benzopyrrolizidine scaffold have not been described as potential antigiardiasic agent. The most closely related compound is flinderole C, a tricyclic derivative containing a benzo-fused pyrrolizidine which exhibits antimalarial activity; however, its mechanism of action remains unknown (Fernandez et al., 2009). Otherwise, benzopyrrolizidines have been also reported to exhibit antioxidant activity and prevent lipoperoxidation (Miranda and Hernández-Vázquez, 2015).

A preliminary structure-activity relationship (SAR) analysis of the benzopyrrolizidine core suggested that two methoxy groups at the arylidene moiety increased the antigiardiasic activity. This result was observed in three benzopyrrolizidines (EHV-110-154, EHV-110-160, and EHV-110-174, **Scheme 1C**) which showed the lowest IC_50_ values against trophozoites (30, 20, and 11 µM, respectively). Substitution of these moieties for other groups reported at the R^2^ position (**Table S1**), limited the maximum antigiardiasic activity yield to 25%, even at the highest concentration assayed (100 µM). Besides, incorporation of a (*S*)-1-phenylethyl in R^1^ provoked the most important antigiardiasic activity while the replacement by a *tert*-butyl or cyclohexyl reduced the effect. The effects after substitution at R^3^ were not clear; however, the addition of a methoxy group at the 7C position increased the percentage of trophozoite death from 8.2% (EHV-110-106) to 30% (EHV-110-135). The same was observed when a fluorine was attached at the 6C (EHV-110-160, 67%). More substitutions on the benzopyrrolizidine scaffold are currently under examination to obtain a more precise SAR analysis.

### Morphologic Studies of Trophozoites

**Figure 1:**
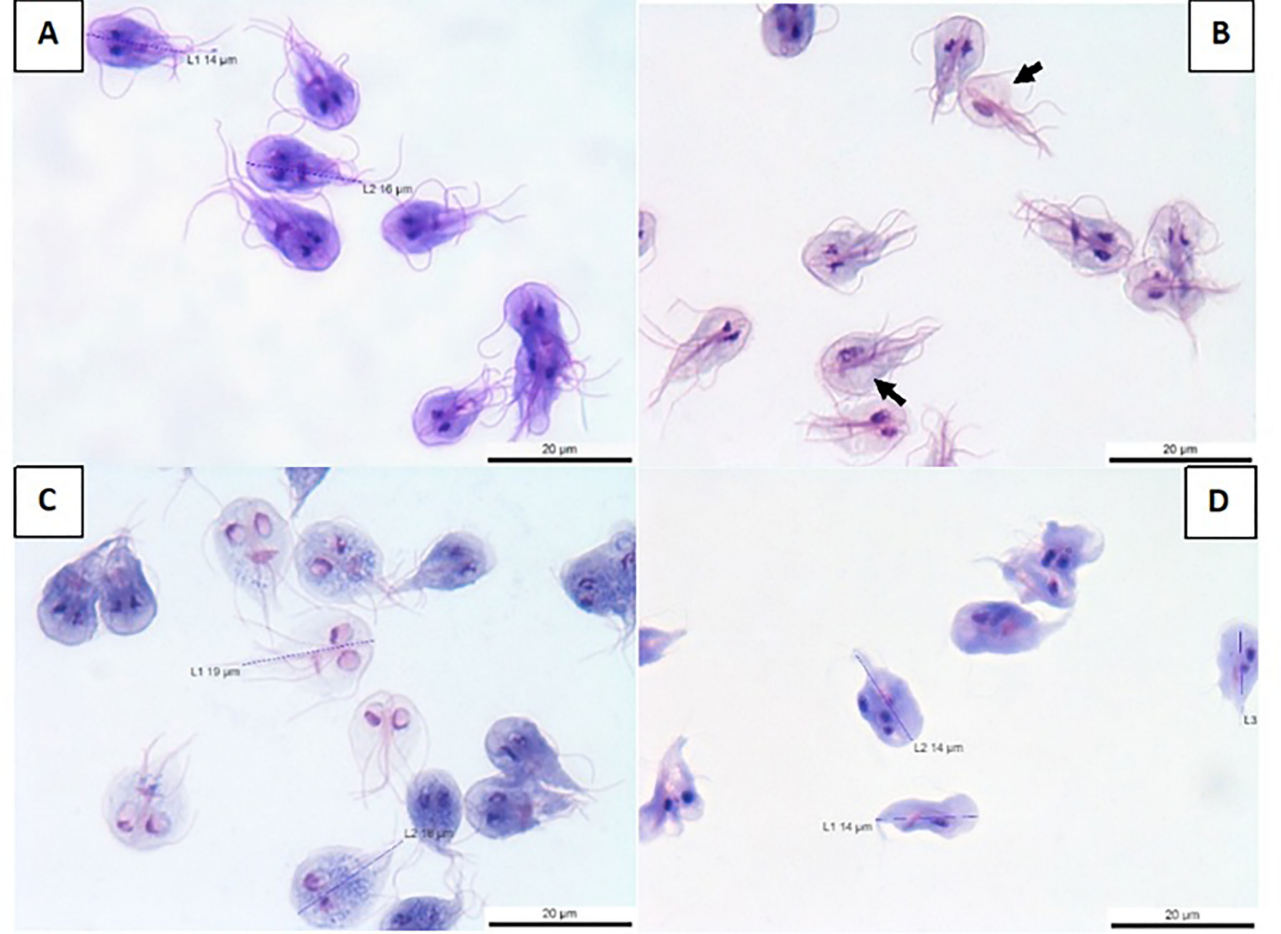
Morphological effects of benzopyrrolizidines on *G. lamblia* trophozoites. **(A)** Control cell sample; **(B)** cells treated with EHV-110-174; **(C)** metronidazole-treated trophozoites; **(D)** albendazole-treated trophozoites. The images have 100X. The arrows show those trophozoites that lost a nucleus.

To shed light on the antigiardiasic mechanism of benzopyrrolizidines, we performed structural and ultrastructural analysis on treated *G. lamblia* trophozoites and compared them versus the standard drugs (metronidazole, tinidazole, and albendazole). **Figure 1A** shows control trophozoites with the classical pear-shaped morphology with four pairs of flagella, a smooth surface, and two well-defined nuclei. On the contrary, **Figure 1B** shows the effect of compound EHV-110-174, where the cells are translucent and swelling, with shortening of flagella but without general morphological deformation. Remarkably, the nuclei seem altered, and in several trophozoites, one of them is lost (see arrows in **Figure 1B**). To confirm that benzopyrrolizidine molecules induce this kind of damage to the nuclei, we evaluated three more analogues: EHV-110-135, EHV-110-154, and EHV-110-160 (**Figures S1A–C**, respectively). In consonance with the lead **EHV-110-174**, this group also affected the nuclear structure and promoted the loss of one nucleus. This evidence strongly suggests that the benzopyrrolizidine core induce a singular damage pattern on cellular nuclei.

Treatments with either nitroimidazole drugs, metronidazole (**Figure 1C**) or tinidazole (**Figure S1D**), provoked cell swelling and the voiding of cytoplasmic contents making the cells translucent. These changes are elicited by the production of free radicals through pyruvate ferredoxin oxidoreductase action with the nitroimidazoles (Leitsch et al., 2011). Albendazole, a benzimidazole derivative, induces tubulin polymerization inhibition which favors the cytoskeleton disorganization of trophozoites (Oxberry et al., 1994). Consequently, an intense deformation of its general structure was observed (**Figure 1D**). Hence, the optical microscopy data point out the disturbances in trophozoite morphology that benzopyrrolizidine molecules caused, compromising both cytoplasm and nuclei, and being strikingly different from the well-defined changes generated by the current drugs, nitroimidazoles, and benzimidazoles.

**Figure 2:**
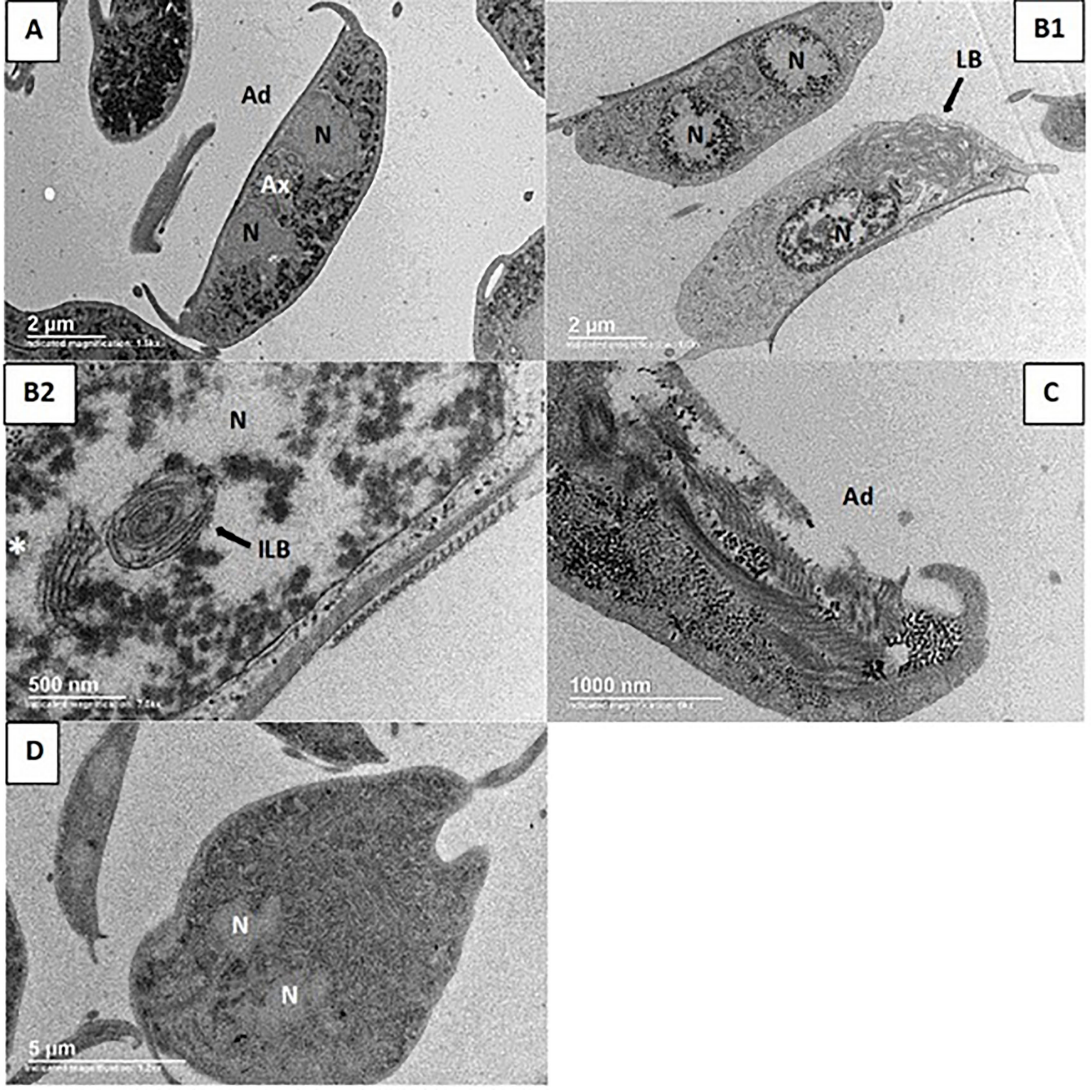
Transmission electron micrographs from *G. lamblia* trophozoites. **(A)** Control of trophozoite. **(B1, B2)** Trophozoites treated with EHV-110-174; in **(B1)**: Trophozoite with its condensed chromatin distributed in the nucleus periphery, the other trophozoite containing a huge lamellar body (LB) covering almost half of the cell. **(B2)**: Close-up image; the nucleus has condensed chromatin and contains an intranuclear LB (ILB) and a cistern-like structure (white asterisk). **(C)** Albendazole-treated trophozoite exhibiting loss of adhesive disc. **(D)** Metronidazole-treated trophozoite without apparent morphologic changes. N, nucleus; Ax, axonemes; Ad, adhesive disc.

The ultrastructural analysis showed that, contrary to control trophozoites whose organelles exhibit normal morphology (**Figure 2A**), the EHV-110-174 treatment damaged the cellular nuclei inducing chromatin condensation and conferring to it a speckled pattern (**Figures 2B1, B2**). We also observed unusual structures resembling lamellar bodies (Wertz, 2018) (LB; see panel B2 and B5). These LB are located in both cytoplasm and nuclei, together with a system of parallel membranes with vesicles-like structure (see structure near of the asterisk, **Figure 2B2**). Further, we want to highlight the loss of the continuity from nuclear envelope, resembling a programmed cell death process (Bagchi et al., 2012).

In summary, the changes with the treatment of the benzopyrrolizidines consisted of cell swelling, morphologic modifications of the nuclei, with occasional loss of one of them, chromatin condensation, shortening of flagella, unusual LB structures in both nuclei and cytoplasm, and loss of the cytoplasmic content, but conserving the general morphology of trophozoites. Not all of these alterations occurred with albendazole or metronidazole treatment (**Figures 2C, D**, respectively). The LB forms have been observed in a broad spectrum of organisms, including mammals, birds, reptiles, and parasites (Matoltsy and Bednarz, 1975; Corrêa et al., 2009; Bagchi et al., 2012; Menon et al., 2018). The presence of LB in the cytoplasm may implicate disorders of lipid metabolism and accumulation of toxic metabolites. Evidence from poisoned birds suggests that LB are necessary for eliminating toxic compounds from these organisms (Menon et al., 2018). In the case of *Giardia*, LB structures have been observed during cell death such as autophagic-like or apoptosis-like processes (Corrêa et al., 2009; Bagchi et al., 2012). Interestingly, a recent work describing the effect of a histone deacetylase inhibitor (named as KH-TFMDI) on *Giardia* trophozoites reported morphological changes including membrane blebbing, chromatin condensation, and lamellar bodies (Gadelha et al., 2019), which are similar to our findings. In this context, a comparison between KH-TFMDI, a 3-arylideneindolin-2-one derivative (Veiga-Santos et al., 2014), and benzopyrrolizidines shows that both molecules share some structural features. Although this comparison might suggest a deacetylase-inhibitory activity as a potential mechanism of drug action for benzopyrrolizidine molecules, additional data must perform to support this proposal.

## Conclusions

The low-throughput screening campaign described herein revealed that benzopyrrolizidine might be a molecular lead for designing new antigiardiasic drugs with high activity and low host toxicity. Furthermore, preliminary data support the idea that its mechanism of action has not been described and suggests a likely new biological target. This circumstance is encouraging, as the target is not subject to selection for resistance mechanisms and avoids potential treatment failure. Thus, we believe that these preliminary results open a new avenue for developing novel therapeutic agents against this parasitosis. Studies are currently underway to discover the mechanism of action and the biological target and the separation and biological evaluation of both diastereomers of EHV-110-174.

## Materials and Methods

### *In Vitro* Cytotoxicity Microassay

The cytotoxicity test was performed using sterile 96 well clear flat bottom polystyrene TC-treated microplates (Corning Costar #3596). Every well contained in a triplicate 287.5 µL medium supplemented with a first set of 54 compounds of the collection, control drugs (metronidazole and tinidazole), and negative controls (0.05% DMSO). Afterwards, 12.5 µL of a cell suspension containing 1.6 x 10^7^ trophozoites/mL was added to every well to obtain a final number of 2x10^5^ cells/well and incubated at 37°C for 24 hours. The final concentration of 54 compounds, metronidazole, and tinidazole was 100 µM; in all cases, DMSO was adjusted to 0.05%. A triplicate of blank was done by filling three wells with only 300 µL culture medium (no cells but DMSO). After that, the plates were chilled for 30 minutes to separate the adhered trophozoites in wells. Later, we transferred 50 µL of this suspension to a second plate containing 250 µL of TYI-S-33 media free of compounds, DMSO or antibiotic per well, and re-cultivated at 37°C for 24 hours. The test was performed by two independent experiments for all compounds.

### Quantification of Cellular Proliferation and Viability

>The trophozoites viability was evaluated using the 2,3-Bis(2-methoxy-4-nitro-5-sulfophenyl)-sH-tetrazolium-5-carboxianilide inner salt (XTT) assay. Antigiardiasic activity of each compound was expressed as the death percentage, calculated by using the next equation (Inc, 2020):


$$\%\;death=\left[\frac{\left(Abs.\;of\;well\;with\;100\%\;viability-Abs.\;of\;well\;with\;compound\right)}{\left(Abs.\;of\;well\;with\;100\%\;viability-Abs.\;of\;blank\;well\right)}\right]X\;100$$

The assay was performed by two independent experiments, where each compound was tested by triplicate.

### Cytotoxicity Assay of Tested Compounds in Human Cells

The *in vitro* cytotoxicity evaluation of compounds was assayed in immortalized human keratinocytes cell line (HaCat). Briefly, cell cultures were grown in DMEM-F12 media supplemented with 10% decomplemented fetal bovine serum, at 37°C and 5% CO_2_. Before to test, cells were harvested by centrifugation at 25°C and resuspended in fresh media at 5x10^4^ cells/mL. Then, the cells were placed in sterile 24 well clear flat bottom polystyrene TC-treated plates (Corning Costar, #353047) at 3x10^4^ cells/well in a volume of 600 µL during 24 hours/37°C at 5% CO_2_, 800 µL of media was replaced by the same amount of fresh media containing the compounds at concentrations of 100, 90, 80, 70, 60, and 50 µM; DMSO was used as a negative control. The cells were incubated for 48 hours/37°C and 5% CO_2_. In the end, the cells were washed two times with PBS, fixed and stained by incubating 15 minutes in a solution containing 50% EtOH, 1.75% formaldehyde, 0.75% crystal violet, and 0.25% NaCl. Then, the plates were washed several times with water until removing the colorant excess. After that, the plates were dried for one day at 25°C. Finally, 600 µL of PBS supplemented with 1% SDS was added to wells. The crystal violet incorporated to cells was spectrophotometrically quantified at 570 nm; the IC_50_ values were calculated from the percentage of death obtained from the different concentrations of assayed compounds. The assay was performed by two independent experiments, each compound was tested by triplicate.

### Morphological Studies of *Giardia* Trophozoites

For bright-field microscopy (BFM), 2x10^6^ trophozoites were placed in 1.5 mL vials containing antibiotic-free TYI-S-33 culture medium, with or without metronidazole, tinidazole, albendazole, or the library compounds, at the IC_50_ concentration values. The trophozoites were incubated for 24 hours/37°C and harvested by chilling in ice bath for 15 minutes and centrifuged at 2,000 x g/5 minutes. The bottom cell was washed three times with PBS, and then, placed in glass slides, fixed with MeOH and stained with Giemsa. Finally, the samples were analyzed by BFM. To identify ultrastructural damage, 1x10^6^ trophozoites treated as described before were fixed with 2.5% glutaraldehyde and post-fixed with 1% osmium tetroxide in 0.1 M cacodylate buffer, pH 7.2. Afterward, the samples were dehydrated with sequential increasing of ethanol concentrations and then, embedded in epoxy resin. Ultrathin sections of samples were analyzed in a JEOL-JEM-1200 transmission electron microscope coupled to a CCD GATAN camera in the Unidad de Imagenología del Instituto de Fisiología Celular, UNAM.

## Data Availability Statement

The original contributions presented in the study are included in the article/**Supplementary Material**. Further inquiries can be directed to the corresponding authors.

## Author Contributions

Conceiving and Conceptualization: HR-V, JO-H, LM, and JA-B. Experimentation, data analysis, and curation: HR-V, JO-H, and LM (data curation and analysis). JA-B, YR-G, JF-R, and LM (cytotoxicity microassay). LM, EH-V, and DG-C (chemistry: synthesis, purification, and molecular 3D-structure). Methodology: JA-B, JF-R, and AT-A. Writing Original draft: HR-V, JO-H, LM, and EH-V. Writing, review and editing: HR-V, JO-H, AC-V, LM, EH-V, MP-M, and MM-G. Supervision, fund acquisition, and project administration: HR-V, AC-V, and MP-M. All authors contributed to the article and approved the submitted version.

## Funding

HR-V and JO-H are supported by Instituto Nacional de Pediatría (INP) grants (No. 2021/006 and 2018/034, respectively). JA-B and JF-R were recipients of fellowships from CONACyT (No. 589503 and 736182, respectively). LM is supported by a grant of CONACyT (No. 284976).

## Conflict of Interest

The authors declare that the research was conducted in the absence of any commercial or financial relationships that could be construed as a potential conflict of interest.

## Publisher’s Note

All claims expressed in this article are solely those of the authors and do not necessarily represent those of their affiliated organizations, or those of the publisher, the editors and the reviewers. Any product that may be evaluated in this article, or claim that may be made by its manufacturer, is not guaranteed or endorsed by the publisher.
