# Supplementary material for: Discovery of Benzopyrrolizidines as Promising Antigiardiasic Agents
Source: Front Cell Infect Microbiol. 2022 Jan 12;11:828100. doi: 10.3389/fcimb.2021.828100 (PMC8790063; doi:10.3389/fcimb.2021.828100)
Supplement: Supplementary file 1 [file DataSheet_1.docx]

**SUPPORTING INFORMATION**

**DISCOVERY OF BENZOPYRROLIZIDINES AS PROMISING ANTIGIARDIASIC AGENTS**

Juan Carlos Auriostigue-Bautista, Eduardo Hernández-Vázquez, Davir González-Calderón, Jorge Luís Figueroa-Romero, Adriana Castillo-Villanueva, Angélica Torres-Arroyo, Martha Ponce-Macotela, Yadira Rufino-González, Mario Martínez-Gordillo, Jesús Oria-Hernández^*^, Horacio Reyes-Vivas^*^ Luis D. Miranda^*^

**Table of content**

| **Table S1.** Structure of benzopyrrolizidine derivatives………………………………………… | **S2** |
| --- | --- |
| **Table S2.** Structure of pyrazinone derivatives………………………………………………… | **S3** |
| **Table S3.** Structure of pyrazinoisoquinoline derivatives……………………………………… | **S4** |
| **Table S4**. Structure of b*is*(aryl-ether) macrocycles…………………………………………… | **S5** |
| **Table S5**. Structure of biphenyl-containing macrocycles…………………………………….. | **S6** |
| **Table S6**. Structures of isoindolones…………………………………………………………… | **S7** |
| **Table S7**. Structure of aliphatic diphenylamines………………………………………………. | **S8** |
| **Table S8.** Antigiardiasic activity of 54 compounds on *Giardia lamblia* trophozoites cultures……………………………………………………………………………………………... | **S9** |
| **Table S9** Confirmatory assay of the better antigiardiasic compounds……………………… | **S10** |
| **Table S10** Antigiardiasic activity in trophozoites from compounds containing the best giardicidal scaffolds identified in the first screening……………………………………………………….. | **S11** |
| Chemical synthesis of **EHV-110-174**…………………………………………………………… | **S12** |
| Spectra of compound **12**………………………………………………………………………… | **S14** |
| Spectra of **EHV-110-174**………………………………………………………………………….. | **S17** |
| **Figure S1** Morphological effects of benzopyrrolizidines on *G. lamblia* trophozoites……….. | **S21** |

**Table S1.** Structure of benzopyrrolizidine derivatives.

|  | | | |
| --- | --- | --- | --- |
| **Compound** | **R^1^** | **R^2^** | **R^3^** |
| EHV-110-108 | *t*-Bu | 4-OH-3-OMe | H |
| EHV-110-139 | *t*-Bu | 4-OH-3,5-diOMe | H |
| EHV-110-154 | Cy | 3,4-diOMe | H |
| EHV-110-160 | *t*-Bu | 3,4-diOMe | 6-F |
| EHV-110-174 | (*S*)-1-phenylethyl | 3,4-diOMe | H |
| EHV-110-178 | 2,6-dimethylphenyl | H | H |
| EHV-110-106 | *t*-Bu | 3,4-diOMe | H |
| EHV-110-135 | *t*-Bu | 3,4-diOMe | 7-OMe |
| EHV-110-146 | *t*-Bu | H | 7-OMe |
| EHV-110-173 | *t*-Bu | 4-OH-3-OMe | 7,8-diOMe |
| EHV-110-178 | *t*-Bu | H | H |

**Table S2.** Structure of pyrazinone derivatives.

|  | | |
| --- | --- | --- |
| **Compound** | **R^1^** | **R^2^** |
| EHV-110-AN-5 | Cyp | 3,4,5-(triOMe)Ph |
| EHV-110-AN-16 | Bn | 3,4,5-(triOMe)Ph |
| EHV-110-AN-24 | *t*-bu | 4-(OH)Ph |
| EHV-110-AN-28 | Bn | 4-(OH),3,4-(diOMe)Ph |
| EHV-110-AN-44 | Cy | 4-(NO_2_)Ph |
| ACR-162-76 | Cy | 4-(F)Ph |
| ACR-162-86 | Cy | Ph |
| ACR-162-92 | Cy | 3,4-(diOMe)Ph |
| ACR-162-94 | Cy | 4-(OMe)Ph |

**Table S3.** Structure of pyrazinoisoquinoline derivatives.

|  | | | |
| --- | --- | --- | --- |
| **Compound** | **R^1^** | **R^2^** | **R^3^** |
| EH-110-5 | Cy | H | H |
| EH-110-6 | *t*-Bu | H | H |
| EH-110-187 | 2,6-dimethylphenyl | H | 9-Me |
| EH-110-188 | 2,6-dimethylphenyl | Me | H |
| EH-110-190 | *t*-Bu | H | 8,9-O_2_CH_2_ |
| EH-110-192 | *t*-Bu | H | 8-F |
| EH-110-198 | Bn | H | H |
| EH-110-212 | 2,6-dimethylphenyl | H | H |
| EH-110-214 | *t*-Bu | H | 9-Me |
| EH-110-4 | (*S*)-1-phenylethyl | H | H |

**Table S4**. Structure of b*is*(aryl-ether) macrocycles.

|  | | | |  |
| --- | --- | --- | --- | --- |
| **Compound** | **R^2^** | **R^2^** | **n** | |
| EHV-110-MA-48 | 4-(OMe)phenethyl | Cy | 1 | |
| EHV-110-MA-50 | 4-(OMe)Bn | 2,6-dimethylphenyl | 1 | |
| EHV-110-MA-70 | 4-(CH_3_)Ph | *t*-Bu | 1 | |
| EHV-110-MA-74 | 4-(*i*Pr)Ph | *t*-Bu | 1 | |
| EHV-110-MA-78 | 4-(*i*Pr)Ph | Cy | 1 | |
| EHV-110-MA-84 | 4-(F)Ph | Cy | 1 | |
| EHV-110-MA-86 | 4-(CH_3_)Ph | Cy | 2 | |
| EHV-110-MA-92 | 4-(CH_3_)Ph | 2,6-dimethylphenyl | 2 | |
| EHV-110-MC-60 | 4-(CH_3_)Ph | Cy | 1 | |
| EHV-110-MC-66 | 4-(CH_3_)Ph | 4-(OMe)Ph | 1 | |
| EHV-110-MC-68 | 4-(CH_3_)Ph | Bn | 1 | |
| EHV-110-MC-70 | 4-(*i*Pr)Ph | Cy | 2 | |
| EHV-110-MC-74 | 4-(F)Ph | Cy | 2 | |
| EHV-110-MC-76 | 4-(CF_3_)Ph | Cy | 2 | |
| EHV-110-MC-78 | Cy | Cy | 1 | |
| EHV-110-MC-80 | 3,4-O2CH_2_ | Cy | 1 | |
| EHV-110-MC-82 | Bn | Cy | 1 | |
| EHV-110-MC-84 | 4-(OMe)Ph | Cy | 1 | |
| EHV-110-MC-86 | Phenethyl | Cy | 1 | |
| EHV-110-MC-35 | 4-(OH)phenethyl | Cy | 1 | |
| EHV-110-MC-40 | Bn | 2,6-dimethylphenyl | 1 | |
| EHV-110-MC-44 | Ph | Cy | 1 | |
| EHV-110-MC-48 | Furan-2-yl | Cy | 1 | |
| EHV-110-MC-50 | 4-(CH_3_)Ph | Cy | 3 | |
| EHV-110-MC-54 | 4-(OMe)Bn | *t-*Bu |  | |

**Table S5**. Structure of biphenyl-containing macrocycles.

|  | | | |  |
| --- | --- | --- | --- | --- |
| **Compound** | **R^1^** | **R^2^** | **n** | |
| ***Type A*** | |  |  | |
| MCM-032 | *t*-Bu | *t*-Bu | 1 | |
| MCM-034 | *t*-Bu | Bn | 1 | |
| MCM-068 | *t*-Bu | *t*-Bu | 3 | |
| MCM-075 | *t*-Bu | Bn | 3 | |
| MCM-081 | *t*-Bu | dodecyl | 1 | |
| MCM-082 | *t*-Bu | dodecyl | 3 | |
| ACR-153-56 | *t*-Bu | Cy | 3 | |
| ACR-153-60 | *t*-Bu | Cyp | 3 | |
| ACR-162-73 | CH_2_COOMe | *t*-Bu | 3 | |
| ***Type B*** | |  |  |  |
| MCM-029 | *t*-Bu | *t*-Bu | 1 | |
| MCM-072 | *t*-Bu | *t*-Bu | 3 | |
| MCM-040 | Dodecyl | *t*-Bu | 1 | |
| MCM-042 | Dodecyl | *t*-Bu | 3 | |

**Table S6**. Structure of isoindolones.

|  | | | |
| --- | --- | --- | --- |
| **Compound** | **R^1^** | **R^2^** | **R^3^** |
| ***Type A*** |  |  |  |
| YAAS-129-70 | *i*-Pr | Bn | - |
| YAAS-129-63 | Me | Cy | - |
| ***Type A*** |  |  |  |
| YAAS-144-232 | Cy | OMe | H |
| YAAS-144-239 | Bn | H | H |
| YAAS-144-263 | Ph | OMe | Cl |

**Table S7**. Structure of aliphatic diphenylamines.

| ****  **** |
| --- |

**Table S8.** Antigiardiasic activity of 54 compounds on G. lamblia trophozoites cultures*.

| **Compound** | **% cell death** | **Compound** | **% cell death** |
| --- | --- | --- | --- |
| EHV-110-5 | 16.68 | ACR-153-56 | 13.47 |
| EHV-110-6 | 12.36 | ACR-153-60 | 4.50 |
| EHV-110-108 | 7.97 | ACR-162-19 | 12.53 |
| EHV-110-139 | 24.9 | ACR-162-73 | 8.55 |
| **EHV-110-154** | **100.0** | ACR-162-76 | 12.02 |
| **EHV-110-160** | **100.0** | ACR-162-86 | 9.95 |
| **EHV-110-174** | **100.0** | ACR-162-92 | 15.44 |
| EHV-110-178 | 17.9 | ACR-162-94 | 8.23 |
| EHV-110-187 | 12.28 | ACR-173-45 | 3.50 |
| EHV-110-188 | 27.84 | ACR-173-46 | 6.81 |
| EHV-110-190 | 1.76 | MCM-029 | 6.62 |
| EHV-110-192 | 11.63 | MCM-032 | 3.21 |
| EHV-110-198 | 7.81 | MCM-034 | 4.74 |
| EHV-110-212 | 6.13 | MCM-040 | 3.05 |
| EHV-110-214 | 13.52 | MCM-042 | 6.12 |
| EHV-110MA-48 | 9.76 | MCM-068 | 5.20 |
| EHV-110MA-50 | 10.64 | MCM-072 | 0.20 |
| EHV-110MA-70 | 22.00 | MCM-075 | 17.60 |
| EHV-110MA-74 | 26.16 | MCM-081 | 10.13 |
| **EHV-110MA-78** | **100** | MCM-082 | 12.78 |
| EHV-110MA-84 | 2.88 | YAAS-129-70 | 8.66 |
| EHV-110MA-86 | 13.9 | YAAS-129-63 | 4.33 |
| EHV-110MA-92 | 16.21 | YAAS-144-232 | 6.17 |
| EHV-110AN-5 | 7.38 | YAAS-144-239 | 7.33 |
| **EHV-110AN-16** | **70.91** | YAAS-144-263 | 5.67 |
| EHV-110AN-24 | 7.57 | EHV-110-4 | 5.46 |
| EHV-110AN-28 | 7.95 | Tinidazole | **100.0** |
| EHV-110AN-44 | 4.68 | Metronidazole | **100.0** |
| DMSO | 5.25 |  |  |
| *All compounds were evaluated at a final concentration of 100 µM by using the cytotoxicity microassay described under Material and Method section. Compounds highlighted in bold showed death yields above of 50%. | | | |

**Table S9**. Confirmatory assay of the better antigiardiasic compounds.

| **Compound** | **First screening** | Hits confirmation | | |
| --- | --- | --- | --- | --- |
|  | % cell death at  100 µM | % cell death at  50 µM | % cell death at  25 µM | % cell death at  12.5 µM |
| **EHV-110-154**^1^ | 100.0 | 94.654 | 10.752 | 4.160 |
| **EHV-110-160**^1^ | 100.0 | 98.178 | 66.901 | 2.874 |
| **EHV-110-174**^1^ | 100.0 | 98.312 | 98.298 | 83.695 |
| **EHV-110MA-78**^2^ | 100.0 | 84.887 | 12.098 | 12.098 |
| **EHV-110AN-16**^3^ | 70.91 | 14.349 | 14.309 | 5.808 |
| Tinidazole | 100.0 | ND | ND | 98.499 |
| Metronidazole | 100.0 | ND | ND | 86.562 |
| DMSO | 5.25 | 7.6969 | ND | ND |

All compounds were evaluated by using the cytotoxicity microassay described

under Material and Method section.

Compounds highlighted in bold exhibited death yields above 50% in Table S8.

ND= Non determined.

^1^: Benzopyrrolizidine

^2^: *Bis*(aryl ether) macrocycle

^3^: Pyrazin-2-one

| **Scaffold** | **Compound** | % cell death | | |
| --- | --- | --- | --- | --- |
|  |  | 100 µM | 50 µM | 25 µM |
| *Benzopyrrolizidine* | **EHV-110-106** | **89.2** | 13.4 | 8.2 |
|  | **EHV-110-135** | **98.8** | **97.5** | 30.2 |
|  | EHV-110-146 | 24.5 | NE | NE |
|  | EHV-110-173 | 7.1 | NE | NE |
| *Triazole bis (aryl ether) macrocycles* | EHV-110-MC-66 | 39.7 | NE | NE |
|  | EHV-110-MC-68 | 14.1 | NE | NE |
|  | **EHV-110-MC-70** | **69.5** | 16.4 | NE |
|  | **EHV-110-MC-74** | **73.0** | **54.0** | NE |
|  | EHV-110-MC-76 | **48.6** | 13.1 | NE |
|  | EHV-110-MC-78 | 4.7 | NE | NE |
|  | EHV-110-MC-80 | 15.3 | NE | NE |
|  | EHV-110-MC-82 | 23.1 | NE | NE |
|  | EHV-110-MC-84 | 5.2 | NE | NE |
|  | EHV-110-MC-86 | 31.0 | NE | NE |
|  | EHV-110-MC-35 | 2.7 | NE | NE |
|  | EHV-110-MC-40 | 21.9 | NE | NE |
|  | EHV-110-MC-44 | 26.0 | NE | NE |
|  | EHV-110-MC-48 | 16.1 | NE | NE |
|  | **EHV-110-MC-50** | **53.7** | 34.9 | NE |
|  | EHV-110-MC-54 | 27.8 | NE | NE |
| Controls | Tinidazole | **100.7** | NE | NE |
|  | Metronidazole | **100.1** | NE | NE |
|  | DMSO | 13.3 | NE | NE |
| ^Compounds highlighted in bold exhibited death yields above 50%.^  ^NE: Non-effect^ | | | | |

**Table S10.** Antigiardiasic activity in trophozoites from compounds containing the best giardicidal scaffolds identified in the first screening.

**Chemical synthesis of EHV-110-174.**

All the chemicals used were acquired from Merck and Sigma-Aldrich Company and were of analytical grade. Flash column chromatography was carried out using SiO_2_ 60 (230–400 mesh). Reactions were monitored by TLC using silica plates 60 F254 aluminium sheets and visualization was done by UV light at 254 nm. Melting point for all the new synthesized compounds was determined using open capillary tubes using Fischer-Johns Scientific melting point apparatus. ^1^H- and ^13^C-NMR spectra were recorded on Bruker Avance 300 MHz; δ in ppm rel. to Me_4_Si as internal standard.

**Procedure for the synthesis of (*S,E*)-*N*-(2-bromobenzyl)-3-(3,4-dimethoxyphenyl)-N-(3-oxo-3-((1-phenylethyl)amino)prop-1-en-2-yl)acrylamide (12)**.

In a round-bottom flask, benzoyloxyacetaldehyde (**10**, 0.433g; 2.6 mmol), 2-bromobenzylamine hydrochloride (**8**, 0.58g; 2.6 mmol) and TEA (0.36 mL; 2.6 mmol) were dissolved in anhydrous methanol (40 mL). The mixture was stirred for 1 h at room temperature under an argon atmosphere. Then, 3,4-Dimethoxycinnamic acid (**11**, 0.55g; 2.6 mmol) was added; after 15 min, (*S*)-(−)-α-methylbenzyl isocyanide (**9**, 0.36 mL; 2.6 mmol) was added and the reaction stirred for 48h at room temperature. The solvent was evaporated under reduced pressure and the residue was dissolved in dichloromethane (80 mL), washed with 10% aqueous NaHCO_3_ (2×30 mL portions), dried with Na_2_SO_4_, and evaporated. The crude product was used without purification in the next step. To a stirred solution of the corresponding acylaminocarboxamide (1.0 g; 1.49 mmol) in dichloromethane (50 mL) were added DBU (0.67 mL; 4.4 mmol) and TEA (0.42mL; 3 mmol). The mixture was vigorously stirred for 24 h at room temperature. Then, the solution was sequentially washed with 0.1 N HCl (2×30 mL portions) followed by a saturated NaCl solution (10 mL). The organic layer was dried with Na_2_SO_4_ and evaporated. The crude dehydroalanine was purified by flash chromatography obtaining 0.66 g (80%) of a brown foamy solid. Rf: 0.5 (Hex/EtOAc 3:7). **^1^H-NMR** (300 MHz, Chloroform-*d*) δ 7.62 (d, *J* = 15.3 Hz, 1H), 7.56 – 6.98 (m, 11H), 6.92 (dd, *J* = 8.3, 1.9 Hz, 1H), 6.85 – 6.63 (m, 3H), 6.52 – 6.26 (m, 2H), 5.41 (s, 1H), 5.11 (d, *J* = 14.7 Hz, 1H), 4.99 (p, *J* = 7.0 Hz, 1H), 4.84 (d, *J* = 14.7 Hz, 1H), 3.82 (s, 3H), 3.73 (s, 3H), 1.26 (d, *J* = 6.8 Hz, 3H). **^13^C-NMR** (75 MHz, CDCl_3_) δ 166.69, 162.51, 151.09, 149.10, 144.66, 142.69, 141.27, 135.77, 133.10, 131.36, 130.02, 129.56, 128.56, 128.36, 127.90, 127.52, 127.24, 126.09, 123.98, 122.67, 114.61, 110.97, 110.06, 55.97, 51.58, 49.37, 21.38. **MS** (DART+) m/z [M+H]^+^: 549; **HRMS** m/z calcd for C_29_H_30_^79^Br_1_N_2_O_4_ [M+H]^+^: 549.13889; found: 549.13951.

**Procedure for the synthesis of** **of 2-((*Z*)-3,4-dimethoxybenzylidene)-3-oxo-*N*-((*S*)-1-phenylethyl)-2,3-dihydro-1*H*-pyrrolo[2,1-*a*]isoindole-9b(5*H*)-carboxamide 1 (EHV-110-174)**.

In a round-bottom flask, the corresponding dehydroalanine (**12**, 0.56g; 1.02 mmol) was dissolved in toluene (20 mL), and Pd(AcO)_2_ (0.045g; 0.2 mmol), PPh_3_ (0.08g; 0.3 mmol), and K_2_CO_3_ (0.28g; 2.0 mmol) were added. The solution was degassed by bubbling argon for 20 min. After that, the mixture was allowed to reflux for 12 h under argon atmosphere. Then, the solvent was evaporated and the resulting crude was diluted with dichloromethane (20 mL). The precipitate was filtered and the filtrate was sequentially washed with water (2×15 mL portions) and with a saturated NaCl solution (2 × 10 mL portions). The organic layer was evaporated and the product was purified by silica gel flash column chromatography. Finally, the compound was recrystallized from ethanol in order to eliminate other traces of impurities. 0.26g (55%) was obtained as a white foamy solid. Rf: 0.4 (Hex/EtOAc 1:1). The compound was obtained as a mixture of inseparable diasteromers.**^1^H-NMR** (300 MHz, Chloroform-*d*) δ 8.07 and 7.97 (s, NH), 7.67–7.52 (m, 2H), 7.31–7.04 (m, 17H), 7.04–6.92 (m, 2H), 6.83 (dd, *J* = 18.4, 8.0 Hz, 2H), 6.69 (dd, *J* = 11.0, 8.4 Hz, 2H), 6.58 (d, *J* = 15.8 Hz, 2H), 5.09–4.82 (m, 4H), 4.40 (d, *J*= 15.3 Hz, 1H), 4.26 (d, *J*=15.3 Hz, 1H), 3.84 (s, 3H), 3.83 (s, 3H), 3.78 (s, 3H), 3.77 (s, 3H), 3.65 (d, *J*=16.3 Hz, 1H), 3.52 (d, *J*=15.9 Hz, 1H), 3.38–3.17 (m, 2H), 1.38 and 1.28 (d, *J* = 7.0 Hz, 3H). **^13^C-NMR** (75 MHz, CDCl_3_) δ 172.10, 171.38, 150.02, 148.39, 142.87, 142.44, 141.03, 140.67, 137.52, 136.75, 136.46, 128.80, 128.71, 128.65, 128.44, 128.40, 127.64, 127.57, 127.39, 127.29, 127.01, 125.88, 125.80, 125.17, 124.99, 124.06, 123.94, 122.57, 122.49, 113.51, 113.34, 110.28, 74.30, 55.94, 55.83, 50.60, 50.39, 48.98, 48.91, 44.89, 43.92, 21.82, 21.62. **MS (DART+)** m/z [M+H]^+^: 469; HRMS m/z calcd for C_29_H_29_N_2_O_4_ [M+H]^+^: 469.21273; found: 469.21396.

**^1^H-NMR of (*S,E*)-*N*-(2-bromobenzyl)-3-(3,4-dimethoxyphenyl)-N-(3-oxo-3-((1-phenylethyl)amino)prop-1-en-2-yl)acrylamide (12).**

**^13^C-NMR of (*S,E*)-*N*-(2-bromobenzyl)-3-(3,4-dimethoxyphenyl)-N-(3-oxo-3-((1-phenylethyl)amino)prop-1-en-2-yl)acrylamide (12).**

**MS-DART of (*S,E*)-*N*-(2-bromobenzyl)-3-(3,4-dimethoxyphenyl)-N-(3-oxo-3-((1-phenylethyl)amino)prop-1-en-2-yl)acrylamide (12).**

**^1^H-NMR of 2-((*Z*)-3,4-dimethoxybenzylidene)-3-oxo-*N*-((*S*)-1-phenylethyl)-2,3-dihydro-1*H*-pyrrolo[2,1-*a*]isoindole-9b(5*H*)-carboxamide (EHV-110-174).**

**^13^C-NMR of 2-((*Z*)-3,4-dimethoxybenzylidene)-3-oxo-*N*-((*S*)-1-phenylethyl)-2,3-dihydro-1*H*-pyrrolo[2,1-*a*]isoindole-9b(5*H*)-carboxamide (EHV-110-174).**

**MS-DART of 2-((*Z*)-3,4-dimethoxybenzylidene)-3-oxo-*N*-((*S*)-1-phenylethyl)-2,3-dihydro-1*H*-pyrrolo[2,1-*a*]isoindole-9b(5*H*)-carboxamide (EHV-110-174).**


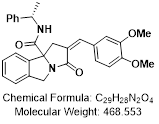

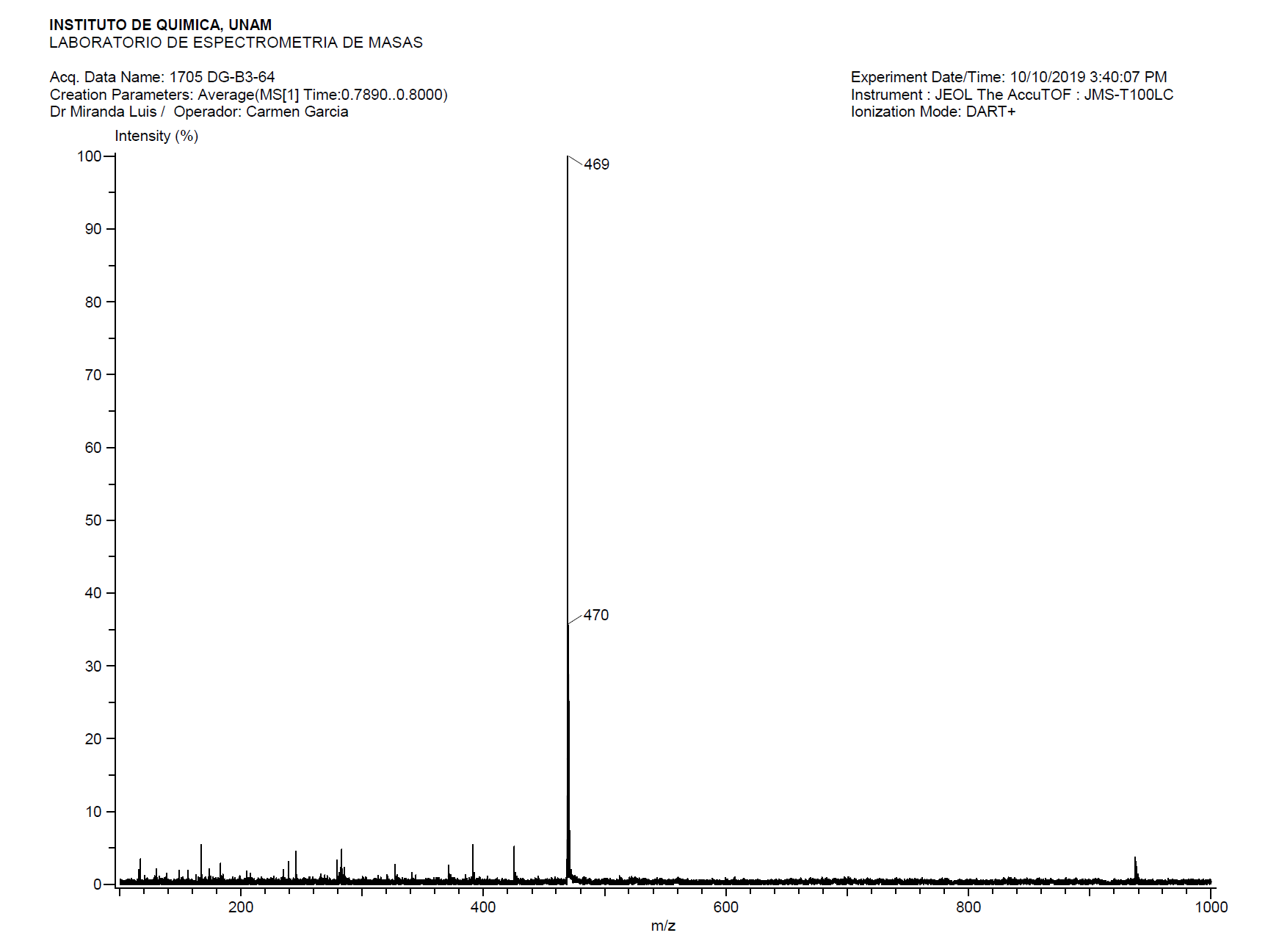
v


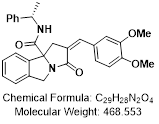
**HRMS of 2-((*Z*)-3,4-dimethoxybenzylidene)-3-oxo-*N*-((*S*)-1-phenylethyl)-2,3-dihydro-1*H*-pyrrolo[2,1-*a*]isoindole-9b(5*H*)-carboxamide (EHV-110-174).**


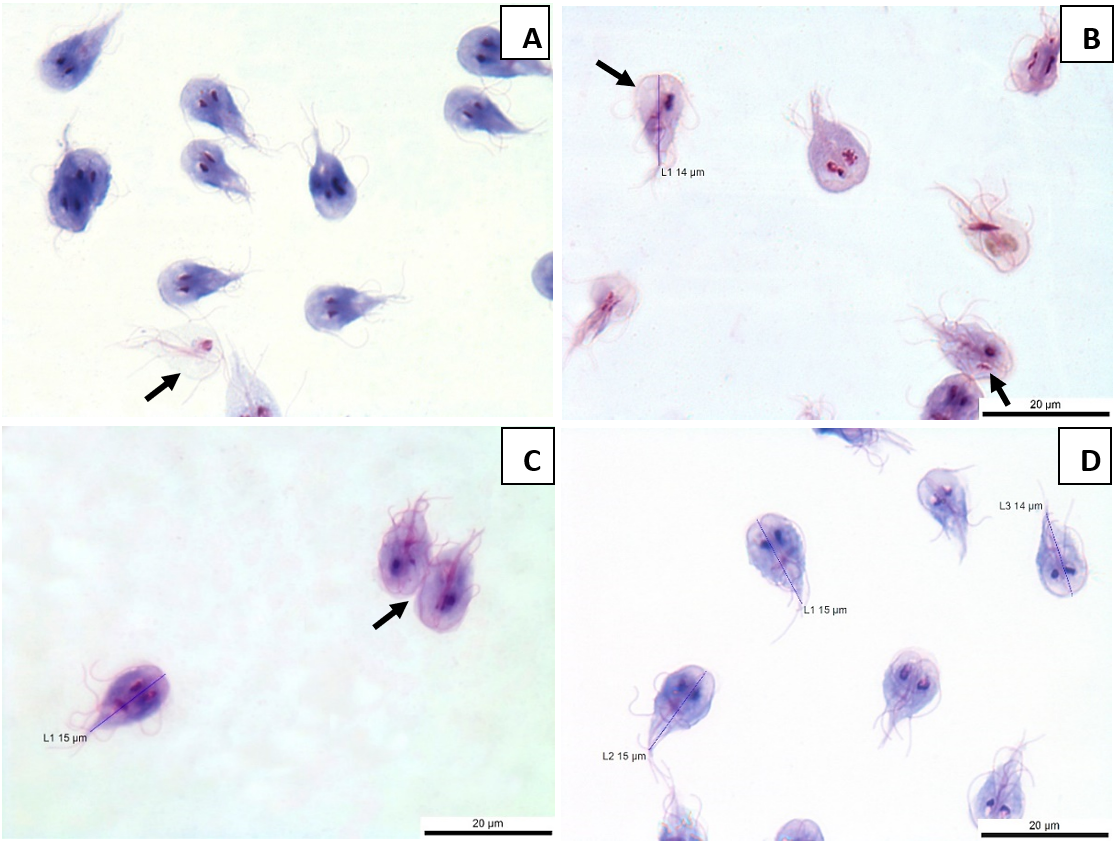


**Figure S1. Morphological effects of benzopyrrolizidines on *G. lamblia* trophozoites**. **A**: cells treated with **EHV-110-135**; **B**: cells treated with **EHV-110-154**; **C**: cells treated with **EHV-110-160**; **D**: cells treated with Tinidazole. The images have 100X magnification. The arrows show those trophozoites that lost a nucleus.
